# Supplementary material for: Modeling glioblastoma heterogeneity as a dynamic network of cell states
Source: Mol Syst Biol. 2021 Sep 16;17(9):e10105. doi: 10.15252/msb.202010105 (PMC8444284; doi:10.15252/msb.202010105)
Supplement: Supplementary file 6 — Source Data for Figure 5 [file MSB-17-e10105-s004.zip › Figure5A_sourcedata/GSEA_3071/nosigdb_stateIII.GseaPreranked.1622795155562/SUVA_AC_PMID_31327527.html]

Details for gene set SUVA\_AC\_PMID\_31327527[GSEA]

|  || Dataset | state33071 |
| Phenotype | NoPhenotypeAvailable |
| Upregulated in class | na\_neg |
| GeneSet | SUVA\_AC\_PMID\_31327527 |
| Enrichment Score (ES) | -0.71902114 |
| Normalized Enrichment Score (NES) | -2.8334188 |
| Nominal p-value | 0.0 |
| FDR q-value | 0.0 |
| FWER p-Value | 0.0 |
Table: GSEA Results Summary

  

Fig 1: Enrichment plot: SUVA\_AC\_PMID\_31327527      
 Profile of the Running ES Score & Positions of GeneSet Members on the Rank Ordered List

  

| PROBE | GENE SYMBOL | GENE\_TITLE | RANK IN GENE LIST | RANK METRIC SCORE | RUNNING ES | CORE ENRICHMENT || 1 | PMP22 |  |  | 1023 | -0.281 | -0.2390 | No |
| 2 | TTYH1 |  |  | 2397 | -0.365 | -0.5608 | No |
| 3 | PRCP |  |  | 2489 | -0.372 | -0.5542 | No |
| 4 | HEY1 |  |  | 2593 | -0.381 | -0.5500 | No |
| 5 | HEPN1 |  |  | 2602 | -0.382 | -0.5215 | No |
| 6 | PCDHGC3 |  |  | 3213 | -0.444 | -0.6418 | No |
| 7 | SLC1A3 |  |  | 3516 | -0.513 | -0.6779 | Yes |
| 8 | TSPAN7 |  |  | 3528 | -0.520 | -0.6390 | Yes |
| 9 | MLC1 |  |  | 3659 | -0.584 | -0.6255 | Yes |
| 10 | F3 |  |  | 3764 | -0.693 | -0.5965 | Yes |
| 11 | SPARC |  |  | 3811 | -0.759 | -0.5475 | Yes |
| 12 | CLU |  |  | 3846 | -0.827 | -0.4900 | Yes |
| 13 | S100B |  |  | 3853 | -0.845 | -0.4238 | Yes |
| 14 | FABP7 |  |  | 3867 | -0.888 | -0.3559 | Yes |
| 15 | PMP2 |  |  | 3876 | -0.959 | -0.2811 | Yes |
| 16 | GPM6B |  |  | 3889 | -1.047 | -0.2003 | Yes |
| 17 | GFAP |  |  | 3907 | -1.280 | -0.1021 | Yes |
| 18 | PTPRZ1 |  |  | 3913 | -1.342 | 0.0041 | Yes |
Table: GSEA details [plain text format]

  

Fig 2: SUVA\_AC\_PMID\_31327527: Random ES distribution      
 Gene set null distribution of ES for **SUVA\_AC\_PMID\_31327527**
